# Scalable entangling gates on ion qubits via structured light addressing

**DOI:** 10.1126/sciadv.aec0392

**Published:** 2026-04-01

**Authors:** Xueying Mai, Liyun Zhang, Qinyang Yu, Junhua Zhang, Yao Lu

**Affiliations:** ^1^ Southern University of Science and Technology, Shenzhen 518055, China.; ^2^ International Quantum Academy, Shenzhen 518048, China.; ^3^ Hefei National Laboratory, Hefei 230088, China.

## Abstract

A central challenge in developing practical quantum processors is maintaining low control complexity while scaling to large numbers of qubits. Trapped-ion systems excel in small-scale operations and support rapid qubit scaling via long-chain architectures. However, their performance in larger systems is hindered by spectral crowding in radial motional modes, a problem that forces reliance on intricate pulse-shaping techniques to maintain gate fidelities. Here, we overcome this challenge by developing a trapped-ion processor with an individual-addressing system that generates steerable Hermite-Gaussian beam arrays. The transversal gradient of these beams couples qubits selectively to sparse axial motional modes, enabling to isolate a single or few modes as entanglement mediator. Leveraging this capability, we demonstrate addressable two-qubit entangling gates in chains up to six ions, with Bell-state preparation fidelities consistently around 0.97, achieved without complex pulse shaping. Our method substantially reduces control overhead while preserving scalability, providing a crucial advance toward practical large-scale trapped-ion quantum computing.

## Teaser

Spatially tailored light offers a simple way to build high-fidelity trapped-ion quantum processors at scale.

## INTRODUCTION

The quest for large-scale quantum computation faces a critical challenge for current trapped-ion systems. While they hold records for gate fidelities and coherence times in small scales (*1*–*4*), both leading architectures in intermediate scale, known as quantum charge-coupled devices (QCCDs) and static large ion crystals (*5*–*9*), exhibit control complexity that grows prohibitively with qubit count (*10*). This escalating overhead threatens to outweigh their quantum advantages. The most severe bottleneck arises in entanglement generation. The reliance on collective motional modes as entanglement mediators creates an intractable conflict: Rapid scaling of qubit counts requires compromising the motional isolation essential for high-fidelity operations (*3*, *4*). This trade-off currently limits the path toward practical large-scale trapped-ion processors.

The core challenge stems from motional mode crowding. In static ion crystals, increasing the ion count raises the density of motional modes, degrading gate fidelity due to uncontrolled residual couplings between qubits and spectator motional modes. QCCD architectures address this by shuttling smaller ion chains but require complicated trap designs with dense electrodes and precise potential control (*11*). Alternatively, while large ion chains enable rapid qubit scaling, they demand increasingly complex pulse sequences to handle the crowded radial motional spectrum to preserving gate fidelity (*12*–*20*), shifting the scaling challenge from hardware design to control system complexity.

While QCCD architectures inevitably face engineering challenges, long ion chains promise a breakthrough by leveraging axial motional modes. The sparse axial mode spectrum enables isolation of single or few modes as entanglement mediators even in hundred-ion chains, substantially simplifying gate operations. However, individual-addressing laser systems necessary for long-chain processors must propagate perpendicular to the ion chain (*21*–*24*), making them incompatible with axial motion couplings. Current implementations of axial-motion–mediated gates are restricted to global entangling operations (*23*, *25*), requiring resource-intensive shelving techniques to entangle arbitrary qubit pairs (*26*). Overcoming these limitation would provide a crucial pathway toward large-scale trapped-ion processors that maintain both high gate fidelity and low control complexity.

Emerging techniques in manipulating spatially structured light suggest potential solutions (*27*, *28*). The electric field profiles of structured light enable engineered light-ion interactions (*29*). For instance, Laguerre-Gaussian beams carrying orbital angular momentum can modify atomic selection rules when interacting trapped ions (*30*, *31*). The transversal profile gradient of the electric field in such beams can couple to all motional df when an ion is positioned at the beam center while simultaneously suppressing the carrier transition (*32*). These unique properties establishes the first viable path toward simultaneously achieving scalable axial-mode coupling and individual qubit control.

In this work, we develope a trapped-ion quantum processor incorporating an individual-addressing system that uses spatially structured Hermite-Gaussian beams. This system enables both precise targeting of arbitrary ion qubits and selective coupling to axial collective motional modes, which is unattainable with conventional Gaussian beam addressing. We demonstrate efficient ground-state cooling and coherent manipulation of axial motion through this tailored light-motion coupling. Building on these capabilities, we implement entangling gates driven by Hermite-Gaussian beams in chains of up to six ions, achieving gate fidelities exceeding 0.97 without complex pulse modulation. Crucially, using both the field gradient and amplitude maxima of the structured light profile, our addressing system can realize a complete universal gate set. These advances establish a crucial step toward scalable trapped-ion processors, substantially reducing the control complexity associated with the long-chain architecture.

## RESULTS

### Experimental setup

**Fig. 1.**
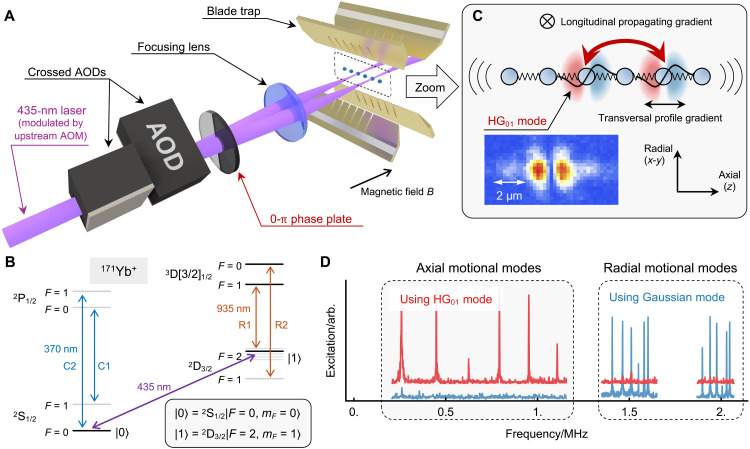
Trapped-ion quantum processor via Hermite-Gaussian light addressing. (**A**) Schematic of the experimental setup. A chain of ^171^Yb^+^ ions is confined in a blade-type ion trap. Individual-addressing beams, propagating perpendicular to the ion chain, are generated using orthogonally oriented acousto-optic deflectors (AODs). A 0-π phase plate after the AODs converts the Gaussian beam into a Hermite-Gaussian (HG_01_) mode at the focal plane. (**B**) Energy levels of single ^171^Yb^+^ ion. Two levels from the ^2^S_1/2_ and ^2^D_3/2_ are used to encode an optical qubit. Transitions driven by 370- and 935-nm lasers are used for state preperation and measurement. (**C**) Spatial gradient of the HG_01_ mode. Conventionl laser beams exhibit longitudinal gradient along the propagating direction. Structured light fields such as the HG_01_ mode introduce an additional gradient arising from sharp spatial variation in field profile. By aligning the profile gradient along the axial direction of the ion chain, we can achieve qubit-qubit coupling mediated by axial motion (parallel to the chain axis). The inset shows the two-dimensional amplitude profile (excluding phase) of the focused HG_01_ mode as sampled by the ion. (**D**) Motional spectrum of a six-ion chain excited by Gaussian and HG_01_ mode beams. For the fundamental Gaussian mode, the propagating vector $\vec{k}$ projects onto both radial directions, exciting two set of radial motional modes in the spectrum. In contrast, the HG_01_ mode used in the experiments selectively excites only axial motional modes. The data of HG_01_ mode are vertically shifted to make it more visible.

We decipt our trapped-ion processor equipped with a Hermite-Gaussian individual-addressing system in Fig. 1A. A chain of ^171^Yb^+^ ions, with a tunable number of ions, is confined in a segmented blade trap. Unlike the conventional scheme that encodes the hyperfine qubit within the ground-state manifold of a single ^171^Yb^+^ ion, we use an optical qubit encoded in both the ground and metastable manifolds, defined as $|0\rangle {=}^{2}S_{1/2}|F=0,m_{F}=0\rangle$ and $|1\rangle {=}^{2}D_{3/2}|F=2,m_{F}=1\rangle$, with an energy gap ω_q_ around 688 THz. This optical qubit is coherently manipulated using a narrow-linewidth 435-nm laser, as shown in Fig. 1B. A magnetic field of ~8 G is generated using permanent magnets, oriented perpendicular to the ion chain and parallel to the propagation direction of the addressing beam. Because the energy gap of our qubit is first-order sensitive to the external magnetic field, a μ-metal shield surrounding the vacuum chamber is used to minimize external magnetic noise. However, the qubit coherence time measured using the 435-nm laser is limited to about 3 ms, primarily due to residual frequency lock noise and fiber phase noise. All qubits can be initialized to the ∣0…0⟩ state via standard optical pumping (*33*). For an optical qubit, its state is measured via state-dependent fluorescence by driving the 370-nm C1 and C2 transitions along with the 935-nm R2 repump transition. Moreover, fluorescence from the ion chain is collected throught an objective lens with 0.4 numerical aperture (NA) and coupled into a multimode fiber array for site-resolved detection. The average detection fidelity for a single ion qubit is around 0.99 (see Materials and Methods for details).

To enable individual addressing of arbitrary ions in the chain, we use a set of crossed acousto-optic deflectors (AODs) to generate and steer an array of laser beams without frequency shift (*34*). An additional fiber acousto-optic modulator (F-AOM), placed upstream of the AODs (not shown in Fig. 1A), is used to rapidly modulate the addressing beams. In contrast to conventional addressing systems, here a spatial mode converter (0-π phase plate) is placed right after the AODs, shaping the focused beam spot into an approximate first-order Hermite-Gaussian (HG_01_) mode. The focusing lens (NA ~ 0.28) is placed on a three-dimensional motorized translation stage to fine-tune the position of addressing beams. As illustrated in Fig. 1C, we characterize the profile of the focused HG_01_ beam using the ion itself, revealing a beam waist of *w*_HG_ = 1.014 (*15*) μm in our setup (see Materials and Methods for details).

Compared to previous demonstrations using Laguerre-Gaussian modes, the Hermite-Gaussian mode can be generated more easily with higher purity (*35*). Moreover, by aligning the dark slit of the HG_01_ mode perpendicular to the ion chain so that the transversal profile gradient is along the axial direction, we can achieve selective coupling to the axial motion of the ions. As shown in Fig. 1D, we can excite all the axial collective motional modes of a six-ion chain using the HG_01_ beam, while conventionally only radial motion can be coupled using fundamental Gaussian mode.

### Manipulating single ion by laser in Hermite-Gaussian mode

**Fig. 2.**
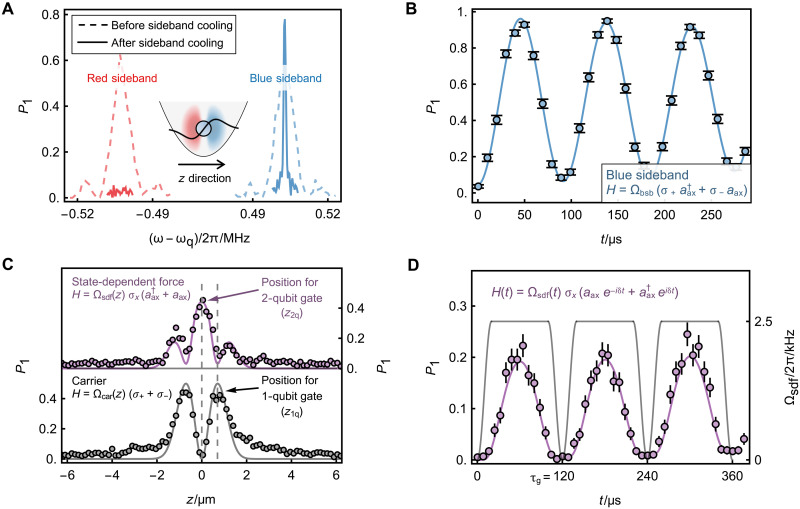
Coherent operations on single ion with laser in Hermite-Gaussian mode. (**A**) Sideband spectrum of axial motion after only Doppler cooling (dotted) and sideband cooling (solid). Sideband cooling suppresses the red sideband transition because the ion mostly occupies the $|0\rangle |0\rangle_{\text{ax}}$ state ($|n\rangle_{\text{ax}}$ denotes axial motional Fock states). (**B**) Coherent blue-sideband oscillation after CSC. The Rabi flopping between $|0\rangle |0\rangle_{\text{ax}}$ and $|1\rangle |1\rangle_{\text{ax}}$ is observed. The fit (solid line) yields coupling strength of Ω_bsb_ = 2π × 5.45 (1) kHz. (**C**) Axial profile of HG_01_-mode laser field properties. After the sideband cooling, the ion is placed along the *z* direction at different locations of the HG_01_ mode. The field gradient is measured via the state-dependent force under the situations of Ω_sdf_(*z* = *z*_2q_) = 2π × 1.6 kHz and the excitation duration τ_p_ = 100 μs. The field amplitude is probed through carrier transition by setting Ω_car_(*z* = *z*_1q_) = 2π × 62.5 kHz and τ_p_ = 2 μs. In our setup, $z_{1q}-z_{2q}\approx 0.7\;\mu m$. The solid lines show numerical simulations assuming a perfect HG_01_ mode profile. (**D**) Qubit state oscillation under detuned state-dependent force. The frequency detuning and maximal coupling strength are set to be δ = 2π × 10 kHz and $\Omega_{\text{sdf}}^{\left(\text{max}\right)}=2\pi \times 2.5$ kHz, respectively. Each 120-μs cycle incorporates 20-μs sin^2^-pulse shaping at both ends. The solid line represents numerical simulation including experimental imperfections. All error bars here and below represent 1σ standard error, and most error bars are smaller than the marker size.

To investigate coherent manipulation of ion qubits using the HG_01_ mode, we begin with a single trapped ^171^Yb^+^ ion, confined with an axial trap frequency of ν_ax_ = 2π × 0.502 MHz. As shown in Fig. 2A, when the ion is placed at the dark slit of the HG_01_ mode, the axial-motion–related sideband transitions (ω_q_ ± ν_ax_) can be both excited after only Doppler cooling. To further cool the axial motion to its ground state, we implement continuous sideband cooling (CSC) via the red-sideband transition driven by the HG_01_-mode laser, assisted by the optical pumping process to pump the ion back to the ∣0⟩ state. In Fig. 2A, we can find that the red-sideband transition is notably suppressed after CSC, indicating near ground-state cooling of the axial motion. Under this situation, the Rabi oscillation of the qubit state under the blue-sideband transition becomes visible, as illustrated in Fig. 2B. The average phonon number estimated from the oscillation is 0.02(2), and the estimation method is described in Materials and Methods.

To implement entangling operations between ion qubits, we required a state-dependent force in the Mølmer-Sørensen gate scheme (*36*, *37*). To examine this force with a single ion, we apply bichromatic frequency components, ω_q_ ± μ (with μ ~ ν_ax_), to the addressing HG_01_-mode laser. It enables engineering the qubit-motion coupling of the form $H=\Omega_{\text{sdf}}\sigma_{x}\left(a_{\text{ax}}e^{i\delta t}+a_{\text{ax}}^{†}e^{-i\delta t}\right)$. Here, σ_*x*,*y*,*z*_ represents the Pauli matrices on single ion qubit, *a*_ax_ ($a_{\text{ax}}^{†}$) is the anihilation (creation) operator on the axial motional mode, Ω_sdf_ is the strength of the qubit-motion coupling, and δ = μ – ν_ax_ is the difference between the trap frequency and the beat-note frequency of the bichromatic fields. Following (*32*), the qubit-motion coupling strength can be rewritten as $\Omega_{\text{sdf}}=\eta_{⊥}\Omega_{\text{car}}^{\left(G\right)}$. Here, $\eta_{⊥}$ is the transversal Lamb-Dicke (tLD) parameter, and $\Omega_{\text{car}}^{\left(G\right)}$ denotes the carrier Rabi frequency when the addressing beam is operated in the fundamental Gaussian mode. In our single-ion setting, the tLD parameter is predicted to be 0.0213(3), while the experimentally measured value is 0.02139(6) (see Materials and Methods for details).

Because the strength of the qubit-motion coupling is propotional to the field gradient of the addressing beam, it should be maximized when the ion is placed at the dark slit of the HG_01_ mode. We verify this by scanning the relative position between the ion and the addressing beam along the axial direction. In this test, the ion is initialized to the ∣0⟩ state and driven by the resonant state-dependent force (δ = 0). The resulting ∣1⟩ state population after excitation, $P_{1}=\left[1-\text{exp}\left(-2\Omega_{\text{sdf}}^{2}\tau_{p}^{2}\right)\right]/2$, is dependent on the coupling strength Ω_sdf_ and the excitation duration τ_p_. By fixing τ_p_, the spatial variant of the gradient can be profiled. As shown in Fig. 2C, the data reveal that the gradient reaches its maximum at the dark slit.

By placing the ion at the dark slit and detuning the state-dependent force from resonance (δ≠0), the qubit periodically decouples from its axial motion while acquiring an additional geometric phase. This phase can lead to entanglment between qubits when multiple ions are driven simultaneously. In Fig. 2D, we show the such periodic oscillation in the measured qubit state population at the experimental setting used in the subsequent entanglment generation.

Moreover, to implement a universal gate set for quantum computing, our addressing system leverages distinct features of the HG_01_ mode for both single- and two-qubit operations. The gradient maxima enable qubit-motion coupling for two-qubit gates, while the amplitude maxima are used for single-qubit rotations. As shown in Fig. 2C, the field amplitude of the addressing beam is characterized by driving the carrier transition. As expected, the excitation probability reaches a minimum at the dark slit, with two symmetric peaks appearing away from the center. The suppression of the carrier transition at the gradient maxima facilitates high-fidelity entangling operations because detrimental effects from parasitic off-resonant couplings are naturally suppressed (*38*). In the following demonstrations, we steer the dark slit of each addressing beam onto the ions to realize two-qubit entangling gates, while one of the intensity peaks is directed onto the ions for single-qubit operations. This steering can be executed dynamically in quantum circuits by adjusting the radio frequencies applied to the AODs. We can also notice that the experimentally measured amplitude profile exhibits a long-tail distribution away from the center. This deviation arises from the intrinsic properties of the approximate HG_01_ mode generated using the 0-π phase plate, as discussed in Materials and Methods.

### Entangling gates in a three-ion chain

**Fig. 3.**
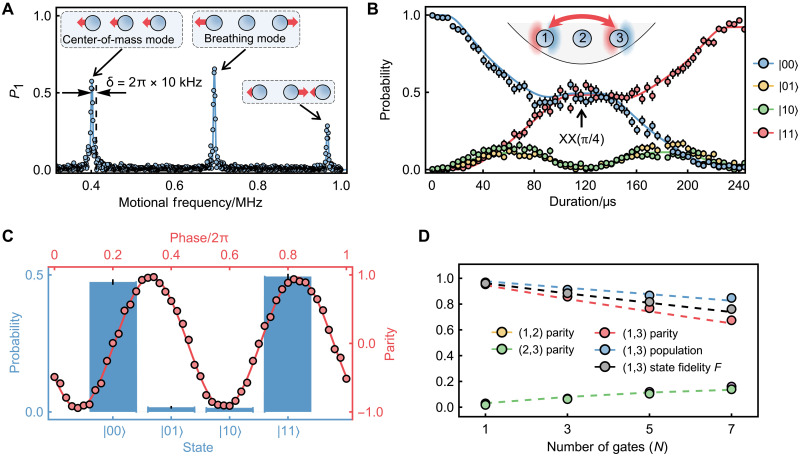
Characterization of two-qubit entangling gates in a three-ion chain. (**A**) Axial motional spectrum of a three-ion chain, showing collective modes at 2π × {0.402, 0.696, 0.967} MHz. The beat-note frequency of the bichromatic fields is detuned by δ = 2π × 10 kHz from the COM mode. All open markers represent experimental data (addressing one outer ion), and the solid line corresponds to numerical simulations. Insets decipt the collective motion patterns. (**B**) Qubit populations during two Mølmer-Sørensen gate cycles for the (1,3) ion pair. All qubits are initialized to ∣0⟩ before operations. Laser power is optimized for ϕ=π/4 at the gate duration of τ_g_ = 120 μs. Colored markers denote experimental results, while solid lines (in corresponding colors) represent numerical simulations incorporating experimental imperfections. (**C**) Bell-state fidelity measurement for the (1,3) ion pair. The blue histogram displays the state populations of the prepared entangled state, while the parity oscillation contrast quantifies off-diagonal coherence. (**D**) Bell-state fidelity versus gate repetitions for the (1,3) ion pair. Colored markers indicate experimental results, and dotted lines show numerical simulations accounting for experimental error sources. Cross-talk–induced entanglement emerges in both (1,2) and (2,3) ion pairs, as evidenced by their increasing parity contrast with successive gate operations.

After characterizing qubit-motion couplings in a single trapped ion, we extend our study to a three-ion chain for implementing entangling gates. The axial trap frequency is relaxed to ν_ax_ = 2π × 0.402 MHz, yielding a nearest-ion spacing of 5.4 μm. As shown in Fig. 3A, three distinct collective motional modes are resolved. We use the center-of-mass (COM) mode for mediating entangling gates due to its uniform coupling across all ion qubits. The beat-note frequency of the bichromatic fields is detuned by 2π × 10 kHz from the COM mode resonance, ensuring negligible coupling to other motional modes. Addressing and applying the state-dependent force to (*j*,*k*) ion pair enables entangling operations, ${\text{XX}}_{j,k}\left(\phi \right)=\text{exp}\left(-\mathrm{i\phi}\sigma_{x}^{\left(j\right)}\sigma_{x}^{\left(k\right)}\right)$, with a gate time of τ_g_ = 120 μs (including 20 μs sin^2^-ramp up/down) according to our settings. In Fig. 3B, we depict the state evolution of (1,3) ion pair under the state-state dependent force. A Bell state can be produced at the gate time by setting ϕ=π/4 via laser power control.

We characterize the Bell-state fidelity by measuring the population of the entangled state and the contrast of the parity oscillation (*39*, *40*). For the (1,3) ion pair, as shown in Fig. 3C, the total population occupied in ∣00⟩ and ∣11⟩ states reaches 0.968(3), with a parity oscillation contrast of 0.953(6), corresponding to a fidelity of 0.960(3). We also generate Bell states for the (1,2) and (2,3) ion pairs, achieving fidelities of 0.949(4) and 0.953(3), respectively (see the Supplementary Materials for details).

To further benchmark gate performance, we measure the decay of the prepared Bell-state fidelity under repeated applications of an odd number of entangling gates. The results for the (1,3) ion pair are summarized in Fig. 3D. Phase-varied π/2-rotations are applied to all three ions to extract parity oscillations of all ion pairs, revealing cross-talk–induced entanglement with the center ion. The measured error rate for the XX_1,3_(π/4) operation is 0.035(1) per gate (noted as per-gate error). The primary error source is dephasing of the 435-nm laser (coherence time around 3 ms as metioned before). Additional contributions include heating of the COM mode (around 250 phonon/s) and limited lifetime of the metastable ^2^D_3/2_ manifold [around 53 ms (*41*)]. The observed increase in parity contrast of (1,2) and (2,3) ion pairs reveals nearest-neighbor gradient cross-talk. Experimental measurements indicate around 1.5% cross-talk, slightly exceeding the theoretical prediction of around 1% (see Materials and Methods for details). We can also notice that the per-gate error is slightly lower than the Bell-state infidelity obtained from single-gate preparation, which may arise from coherent errors or non-Markovian noise in our system. A complete error budget is provided in Table 1. The numerically simulated gate error agrees well with the experimental result. Notably, because we cool the axial motion to its ground state, the effective pointing errors that typically arise in conventional addressing systems due to thermal axial motion are greatly suppressed in our setup (*42*).

**Table 1.** Error budget for the entangling gate XX_1,3_(π/4). The left column summarizes the error sources considered to contribute to the overall gate error. The right column presents numerically estimated contributions, simulated using independently characterized strengths for each error channel. The experimental Bell-state infidelity generated by a single entangling-gate operation is obtained from Fig. 3C. The per-gate error, extracted by fitting the slope of the Bell-state fidelity decay under repeated applications of the entangling gate, is obtained from Fig. 3D.

Error source	Simulated error
Laser dephasing	2.2 × 10^−2^
Heating of COM mode	1.1 × 10^−2^
Gradient cross-talk	0.4 × 10^−2^
^2^D_3/2_ lifetime	0.2 × 10^−2^
Pointing fluctuation	< 1 × 10^−3^
Spectator modes	< 1 × 10^−6^
Simulation sum	3.9 × 10^−2^
Experimental Bell-state infidelity	4.0(3) × 10^−2^
Experimental per-gate error	3.5(1) × 10^−2^

We further validate our error model using the breathing mode to mediate the entangling gate for the (1,3) ion pair. In this configuration, gate errors arising from motional heating and gradient cross-talk are substantially suppressed. Experimental results demonstrate a per-gate error of 0.028(1), in agreement with numerical simulations predicting 0.027 (see the Supplementary Materials for details).

### Extending to longer chains

To further assess the scalability of the HG_01_-driven entangling gates, we extended the system to up to six ions. As in the three-ion case, we use the COM mode to mediate the entangling gates. Because all ions couple uniformly to the COM mode, we only benchmark gate performance on the outermost ion pair (1,*N*), where *N* represents the length of ion chain. These demonstrations also show the advantage of long-range connectivity in trapped-ion quantum processors.

**Fig. 4.**
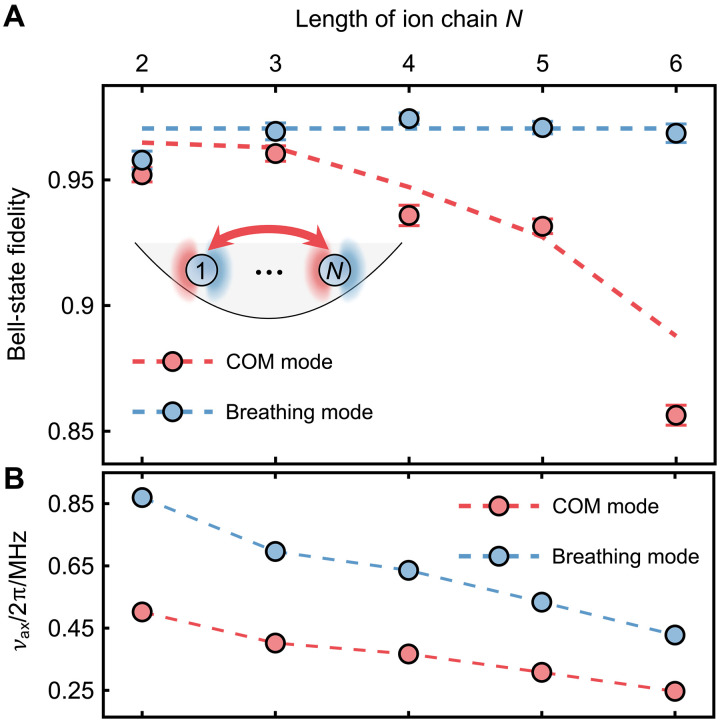
Entanglement fidelities scaling with ion-chain length. (**A**) Measured Bell-state fidelities for outermost ion pairs versus chain length, comparing COM mode (red)– and breathing mode (blue)–mediated gates. All colored markers represent experimental results, and dotted lines are obtain from numerical simulations including experimental imperfections. The Bell-state fidelities for COM-mode–mediated gates are 0.952(3), 0.960(3), 0.936(4), 0.932(3), 0.856(4) for two- to six-ion chains, respectively. While for breathing-mode–mediated gates, the Bell-state fidelities for two to six ions are 0.958(4), 0.969(3), 0.974(4), 0.971(3), 0.969(4), respectively. (**B**) Axial trap frequencies for different chain lengths. For ion chains consisting of two to six ions, we set the axial frequencies to 2π × {0.502, 0.402, 0.367, 0.308, 0.247} MHz (red), and the corresponding breathing-mode frequencies are 2π × {0.869, 0.696, 0.636, 0.533, 0.428} MHz (blue). The uncertainties of all measured trap frequencies are less than 1 kHz.

As shown in Fig. 4A, we measure the fidelity of prepared Bell states as the ion chain length increases. The fidelity remains above 0.95 for two- and three-ion chains, decreased to 0.93 for both four- and five-ion cases, and further dropped to 0.86 for the six ions. Meanwhile, as depicted in Fig. 4B, the axial trap frequency is also lowered down from 2π × 0.502 MHz (two ions) to 2π × 0.247 MHz (six ions) to maintain nearly constant ion spacing. The observed fidelity degradation likely stems from increased motional heating, given the exponential scaling of heating rates with decreasing trap frequency. Thus, we independently measure heating rates at different trap frequencies and include them into numerical simulations. The simulated fidelity trends show agreement with experimental data, except for a deviation in the six-ion case. We attribute this deviation to imperfect ground-state cooling due to the quite large heating rates (around 1600 phonon/s), which amplifies the motion-related errors.

To further verify and mitigate heating impact, we implement entangling gates via the breathing mode, which exhibits a much lower heating rate (less than 10 phonons/s). In this case, we observe consistently Bell-state fidelities of 0.97 for chains up to six ions. The residual errors are primarily attributed to laser dephasing noise and the finite lifetime of the ^2^D_3/2_ state, as discussed in the three-ion case.

Heating-induced gate errors present an inevitable challenge in trapped-ion quantum processors. Here, we demonstrate one mitigation strategy using non-COM motional modes as entanglement mediators. Alternative possible approaches include quantum control to achieve heating-resilient gates (*43*–*45*), as well as cryogenic systems to suppress heating rates by several orders of magnitude (*46*).

We also find that the Bell-state fidelity in the two-ion chain is slightly lower than expected with either mode as mediator. We attribute this extra error to off-resonant carrier coupling caused by the long-tail distribution of the amplitude profile in the HG_01_ mode produced by the phase plate. The same fidelity degradation occurs when entangling adjacent ion pairs in the three-ion chain, suggesting the same reason.

### Scalability

Although isolating a single axial motional mode enables entangling gates without complex pulse modulation at small scale, mode crowding in the axial spectrum becomes unavoidable as the system grows. Maintaining an averaging ion spacing of roughly 3 to 6 μm requires lowering the axial trap frequency as the length of ion chain *N* increases, which, in turn, packs the axial modes closer together. This raises a crucial question for scalability: Can axial-motion–mediated gates continue to provide an advantage as the chain extends to tens or even hundreds of ions?

**Fig. 5.**
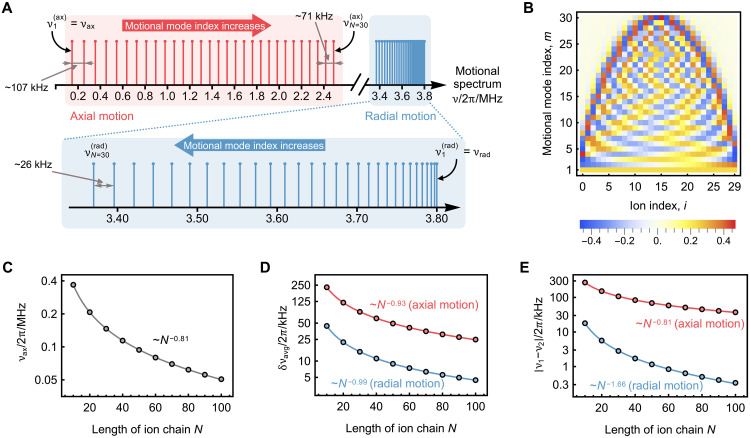
Numerical simulations of motional spectrum properties for ion chains of varying length. (**A**) Collective motional spectrum along the axial and radial directions for a chain of 30 ^171^Yb^+^ ions. The axial and radial trap frequencies are set to ν_ax_ = 2π × 0.146 MHz and ν_rad_ = 2π × 3.8 MHz, respectively. For axial motion, the largest mode separation is ~2π × 107 kHz (near the axial COM mode), while the smallest separation is about 2π × 71 kHz (farther from the COM mode). For radial motion, the largest mode separation is roughly 2π × 26 kHz (away from the radial COM mode). (**B**) Normalized mode-participation coefficients describing the coupling of each ion to each collective motional mode. These coefficients are identical for axial and radial modes that share the same index. (**C**) Scaling of axial trap frequency with ion-chain length. The minimal ion spacing is fixed at 3 μm. All the markers are results from numerical simulation, and the solid line shows the fitted trend. (**D** and **E**) Scaling of the average mode separation (D) and the separation between the first two collective motional modes (E). The red and blue markers correspond to numerical results for the axial and radial motion, respectively, and the solid lines in the matching colors show the fitted trends.

As a specific example, we consider a chain of 30 ^171^Yb^+^ ions, comparable to the state-of-art long–ion-chain quantum processor demonstrated in (*47*). Following the parameters used in that work, we set the axial trap frequency to 2π × 0.146 MHz to maintain a minimal ion spacing of 3 μm, together with a radial trap frequency of 2π × 3.8 MHz. In Fig. 5A, we show the numerically calculcated spectrum of the axial and radial collective motional modes. The average mode speration, defined as ${\delta \nu }_{\text{avg}}=|\nu_{1}-\nu_{N}|/\left(N-1\right)$, is approximately five times larger for the axial modes than the radial modes. Using an entangling-gate time of 200 μs, the required state-dependent force strength to implement an XX(π/4) gate is Ω_sdf_ = 2π × 1.25 kHz. This value is nearly 50 times smaller than the miminal axial mode spacing. Thus, single-mode isolation remains valid even for a 30-ion chain, and entangling gates between any qubit pair can be implemented by selecting an appropriate axial non-COM mode as the mediator.

We also note that, for the axial motion, modes near the COM mode are more sparser than those farther away, whereas the radial spectrum shows the opposite trend. In Fig. 5B, we plot the participation coefficients of all ions in each motional mode. These motional modes near the COM mode involve nearly the entire ion chain, while modes farther from the COM mode couple efficiently only to ions near the center. Consequently, the relatively sparse spectrum near the axial COM mode facilitates the use of axial motion to implement long-range entangling gates.

We further investigate the motional-spectrum properties for chains of up to 100 ions. When the minimum ion spacing is held constant (3 μm in our numerical simulations), the axial trap frequency decreases approximately as $O\left(N^{-0.81}\right)$, reaching about 2π × 0.051 MHz at *N* = 100, as shown in Fig. 5C. The average mode spacing δν_avg_, shown in Fig. 5D, scales as $O\left(N^{-0.93}\right)$ for axial motion and as $O\left(N^{-0.99}\right)$ for radial motion. Despite this reduction, the axial δν_avg_ remains above 2π × 20 kHz up to 100 ions, whereas the radial value has already decreased to around 2π × 4 kHz. The mode speration near the axial COM mode decreases much more slowly than its radial counterpart (Fig. 5E). Our numerical simulations for a 100-ion chain show that, when a non-COM mode is chosen as the entangling mediator, only about three additional modes need to be included in pulse optimization to realize entangling gates between arbitrary qubits. The cumulative error from all other spectator modes can be suppressed below 10^−3^ while keeping the gate time near 200 μs (see the Supplementary Materials for details). This advantage becomes even more pronounced for lighter ion species, such as Ca^+^ and Be^+^, whose higher axial trap frequencies (for the same ion spacing) further increase the mode separations, enabling control of ion chains with hundreds of ions. These long chains can be naturally integrated into QCCD or distributed architectures (*48*) to enable further scaling.

## DISCUSSION

In summary, we demonstrated an individual-addressing system that steers Hermite-Gaussian beam arrays to coherently manipulate and entangle ion qubits. By harnessing the transvesal profile gradient of the Hermite-Gaussian beam, we successfully use the sparser axial motional modes to mediate entangling operations, eliminating the need for complex pulse modulation. This approach substantially reduces control complexity compared to conventional long-chain architectures. In chains up to six ions, we achieve the entangling gate fidelity exceeding 0.97, primarily limited by technical noise sources that can be systematically addressed. Our study provides a scalable pathway for trapped-ion quantum processors that enables efficient scaling to larger qubit numbers while minimizing control overhead.

While we demonstrate entangling gates using optical qubits with Hermite-Gaussian beams, this approach is directly applicable to hyperfine qubits. For hyperfine qubits, one Raman beam should be shaped into the HG_01_ mode, while the other remains in the fundamental Gaussian mode. Crucially, because the gradient for qubit-motion coupling originates from the spatial amplitude profile rather than beam propagation direction, both Raman beams can copropagate. This configuration could notably suppress optical path fluctuation noise.

In our current implementation, the approximate HG_01_ mode generated from 0-π phase plate inherently exhibits nonnegligible cross-talk. For future improvement, alternative spatial mode-shaping techniques such as spatial light modulators, digital micromirror devices (*49*, *50*), or laser-written waveguides (*51*) could be explored to generate high-purity HG_01_ modes for low–cross-talk addressing.

The generation of high-purity HG_01_ modes also provides an alternative approach for breaking the entangling gate speed limit, as parasitic off-resonant carrier couplings in the Mølmer-Sørensen interaction are strongly suppressed, analogous to standing-wave methods demonstrated in previous work (*52*–*54*). Nevertheless, in the fast-gate regime, optimal control remains necessary, as all motional modes are strongly excited, although the suppression of parasitic carrier transitions may help simplify the optimization. Furthermore, by simultaneously coupling multiple ion pairs to different axial motional modes, it is possible to implement parallel entangling gates with minimal control complexity (*55*, *56*). This scheme requires bichromatic fields with different beat-note frequencies to each addressing beams; thus, it is particularly suitable for multichannel AOM-based addressing systems (*21*, *22*).

Future research could also explore interactions with other forms of structured light. For instance, second-order Laguerre-Gaussian beams could enable purely dispersive qubit-motion coupling (*57*), serving for single-shot phonon-resolved measurement in phonon networks (*58*, *59*). The flexibility in engineering diverse qubit-motion interactions may open pathways for developing programmable hybrid quantum simulators incorporating both discrete and continuous variables (*60*–*62*). Recent studies have also shown that polarization gradients in tightly focused Gaussian beams can be exploited to tailor qubit-motion couplings (*63*). These advances would further expand the toolbox of trapped-ion systems for practical quantum applications.

## MATERIALS AND METHODS

### Detection scheme for optical qubit

**Fig. 6.**
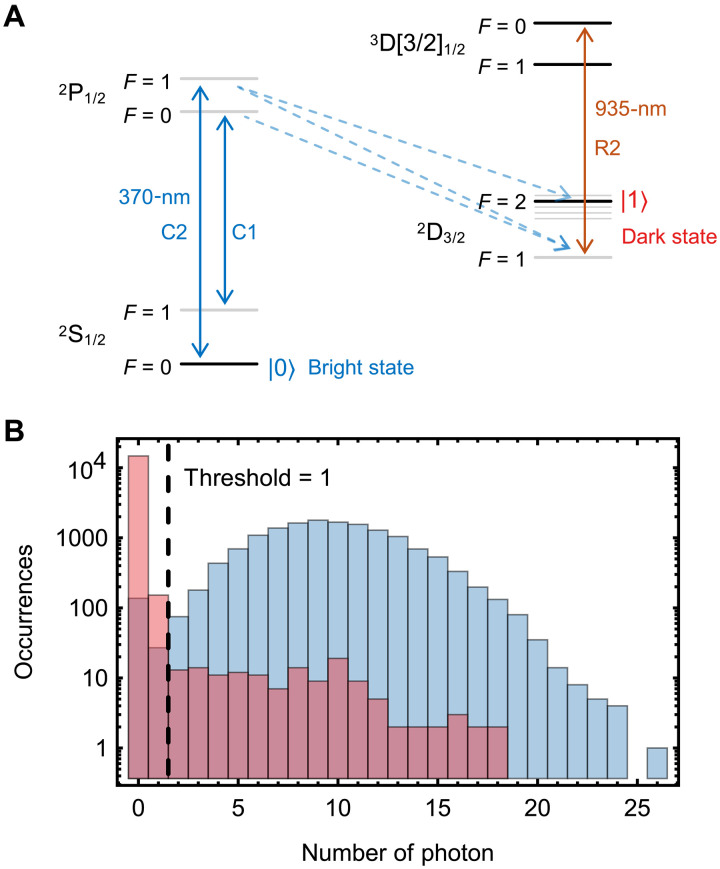
Detection scheme for ^171^Yb^+^ optical qubit. (**A**) Energy levels and transitions for detection process. We use the 370-nm (C1 and C2) and 935-nm (R2) transitions for state-dependent fluorescence detection. The qubit state ∣0⟩ (bright state) generates strong photon scattering, while ∣1⟩ (dark state) remains nonfluorescent due to off-resonant to all detection lasers. (**B**) Photon count statistics. Histograms show photon counts from 15,000 experimental repetitions for states ∣0⟩ (blue) and ∣1⟩ (red). Setting a discrimination threshold at 1 photon yields bright- and dark-state fidelities of 0.9891(8) and 0.9909(8) respectively, resulting in a total detection fidelity of 0.9900(6).

The detection scheme for the ^171^Yb^+^ optical qubit is a variant of the electronic shelving method. As shown in Fig. 6A, the optical qubit state $|0\rangle {=}^{2}S_{1/2}|F=0\rangle$ participates in a closed detection cycle as a bright state, while the ∣1⟩ state within the ${}^{2}D_{3/2}|F=2\rangle$ levels left dark. The main detection cycle is driven by the 370-nm C1 transition and enclosed by the 935-nm R2 transition. The 370-nm C2 transition is additionally driven to pump the ∣0⟩ state population into the detection cycle, making it a bright state. In Fig. 6B, we illustrate photon statistics for both qubit states. The detection fidelity for a single ion is measured to be 0.9900(6). For multi-ion detection, we use a multimode fiber array to simultaneously collect fluorescence across the ion chain. This configuration maintains the detection fidelity of 0.99 for up to six ions with negligible cross-talk.

On the basis of the above precalibrated detection errors, we apply postprocessing error correction to remove detection errors (*64*). The raw detection results and detection error matrix are represented as $P^{\text{meas}}=\left\{p_{0...0}^{\text{meas}},\ldots ,p_{1...1}^{\text{meas}}\right\}$ and **M**, respectively. The true-state populations **P**^real^ are estimated by solving the least-squares minimization problem

$$\text{min}∥P^{\text{meas}}-M.P^{\text{real}}{∥}_{2}$$ (1)

where $∥\cdot {∥}_{2}$ denotes the 2-norm. Given that the detection errors are consistent across all ion qubits and detection cross-talk is negligible, the *N*-qubit error matrix can be expressed as the tensor product of single-qubit error matrices **M**_1q_

$$M=M_{1}⊗\ldots ⊗M_{N}=M_{1q}^{⊗N}$$ (2)

### Generation of Hermite-Gaussian mode

The ideal HG_01_ mode has an electric field distribution of

$$\begin{array}{c} E_{{\text{HG}}_{01}}\left(z\right)=E_{0}\;\times \;H_{1}\left(\sqrt{2}\frac{z}{w_{\text{HG}}}\right)\;\text{exp}\;\left(-\frac{z^{2}}{w_{\text{HG}}^{2}}\right)\\ =E_{0}\frac{2\sqrt{2}z}{w_{\text{HG}}}\;\text{exp}\;\left(-\frac{z^{2}}{w_{\text{HG}}^{2}}\right) \end{array}$$ (3)

where H_1_(*x*) is the first-order Hermite polynomials and *w*_HG_ is the beam waist. The transversal profile gradient can be expressed as

$$\partial_{z}E_{{\text{HG}}_{01}}\left(z\right)=E_{0}\frac{2\sqrt{2}\left(w_{\text{HG}}^{2}-2z^{2}\right)}{w_{\text{HG}}^{3}}\;\text{exp}\;\left(-\frac{z^{2}}{w_{\text{HG}}^{2}}\right)$$ (4)

**Fig. 7.**
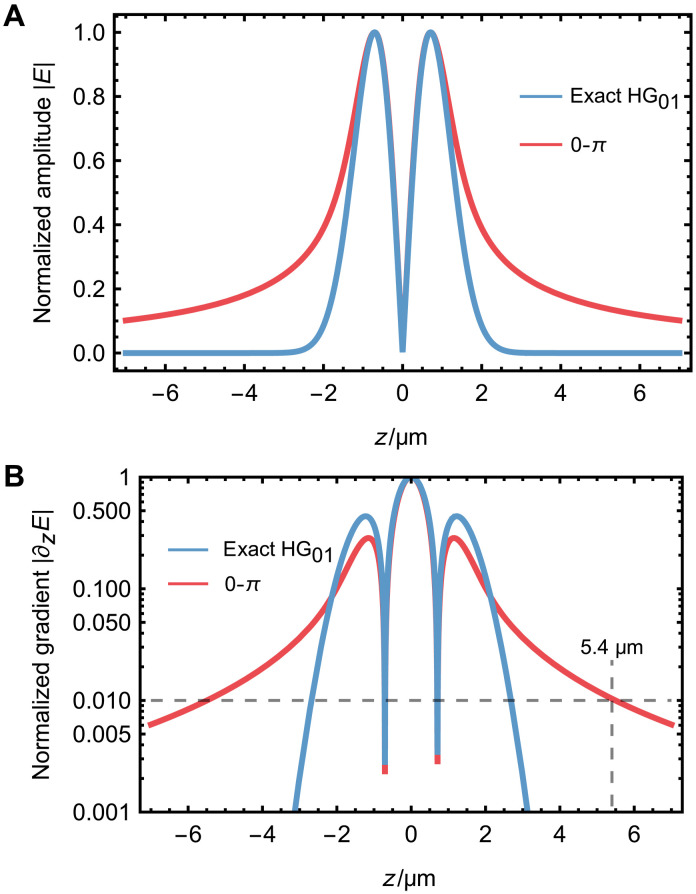
Comparison between exact HG_01_ mode and 0-π phase plate generated structured mode. (**A**) Electric field amplitude profiles normalized to unity peak value. Normalized amplitude distribution ∣E(z)∣ for both the HG_01_ and 0-π mode shows double-peak features. The distance between two peaks is around 1.4 μm, corresponding to the experimental setting. (**B**) Electric field gradient profiles, $|\partial_{z}E\left(z\right)|$, with maximal gradients normalized to unity. The 0-π mode exhibits around 1% gradient cross-talk at 5.4 μm from the center.

In our demonstration, we use a 0-π spatial mode convertor to transform the input Gassian mode into an approximate HG_01_ mode. However, the generated structured mode (referred to as the 0-π mode) exhibits slight deviations from an ideal HG_01_ mode. As shown in Fig. 7A, we compare their electric field amplitude profiles along the *z* direction. We can find that the 0-π mode displays a long-tail distribution compared to the HG_01_ mode, which is also experimentally confirmed, as shown in Fig. 2C.

This long-tail distribution in the amplitude profile also results in a corresponding long-tail in the gradient profile, as shown in Fig. 7B. In our three-ion chain configuration, the spacing of the nearest ion is around 5.4 μm. The intrinsic gradient cross-talk of the 0-π mode is around 1%, while our expeirmental meansurments yield around 1.5%.

### tLD parameter of Hermite-Gaussian mode

Analogous to (*32*), the strength of the sideband interactions is determined by the tLD parameter, defined as

$$\eta_{⊥}\equiv \;\frac{2\sqrt{2}}{w_{\text{HG}}}\;\Delta z$$ (5)

for an HG_01_ beam. Here, $\Delta z=\sqrt{\hbar /2m_{\text{Yb}}\nu_{\text{ax}}}$ denotes the spatial size of the wave packet along the axial direction when the trapped ion is in its motional ground state. Using the tLD parameter, the electric-field profile of the first-order Hermite-Gaussian mode shown in Eq. 3 can be expressed as

$$E_{{\text{HG}}_{01}}\left(z\right)=\frac{\eta_{⊥}z}{\Delta z}E_{G}\left(z\right)$$ (6)

where $E_{G}\left(z\right)=E_{0}\text{exp}\left(-z^{2}/w_{\text{HG}}^{2}\right)$ is the electric-field profile of the fundamental Gaussian mode with the same waist *w*_HG_. Meanwhile, we have to notice that for $E_{{\text{HG}}_{01}}\left(z\right)$ and $E_{G}\left(z\right)$, they satisfy the following relation

$$\int_{-\infty }^{+\infty }{|E_{{\text{HG}}_{01}}\left(z\right)|}^{2}\;dz=2\int_{-\infty }^{+\infty }{|E_{G}\left(z\right)|}^{2}\;dz$$ (7)

In our single-ion setting, the HG_01_ beam waist of *w*_HG_ is 1.014(15) μm, and the axial ground-state wave packet is around 7.6 nm at an axial trap frequency of ν_ax_ = 2π × 0.502 MHz. These values yield a predicted tLD parameter of $\eta_{⊥}^{\left(\text{pred}\right)}=0.0213\left(3\right)$.

To experimentally determine the tLD parameter, one would, in principle, measure the sideband Rabi frequency (Ω_sb_) when the ion is driven by the HG_01_ mode and the carrier Rabi frequency ($\Omega_{\text{car}}^{\left(G\right)}$) when it is illuminated by the fundamental Gaussian mode, using the same beam waist. The optical power of the Hermite-Gaussian beam should be twice that of the fundamental Gaussian beam, as required by the relation in Eq. 7. According to relation in Eq. 6, the tLD parameter can then be calculated from the ratio,

$$\eta_{⊥}=\frac{\Omega_{\text{sb}}\left(z=0\right)}{\Omega_{\text{car}}^{\left(G\right)}\left(z=0\right)}$$ (8)

In our demonstration, we simplify the above procedure using only the HG_01_ mode. In detail, the sideband Rabi frequency (Ω_sb_) is still obtained by steering the dark slit of the HG_01_ mode onto the ion. In contrast, the carrier Rabi frequency (Ω_car_) is measured with the axial center of the addressing beam shifted by $w_{\text{HG}}/\sqrt{2}$ from the ion. Here, the tLD parameter can be obtained from the ratio

$$\eta_{⊥}=\frac{2}{\sqrt{e}}\;\times \;\frac{\Omega_{\text{sb}}\;\left(z=0\right)}{\Omega_{\text{car}}\;\left(z=w_{\text{HG}}/\sqrt{2}\right)}$$ (9)

**Fig. 8.**
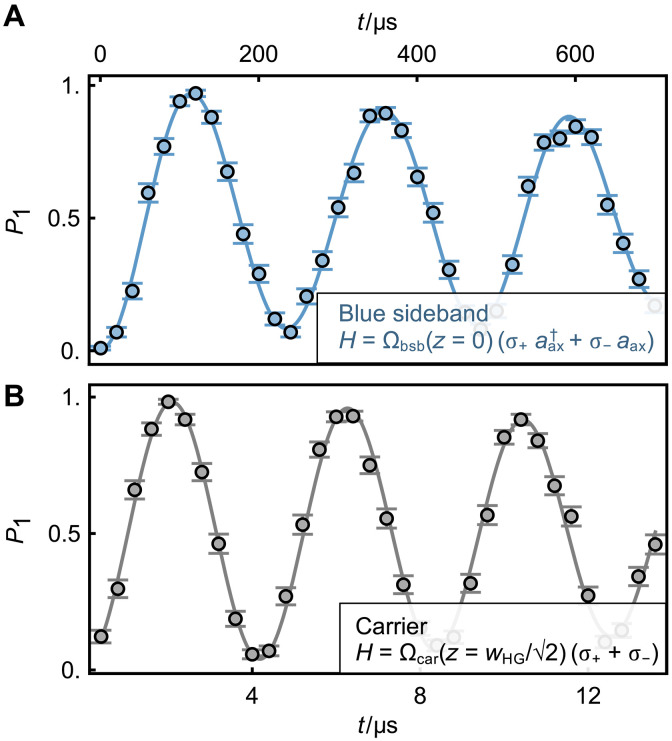
Coherent transitions driven by HG_01_ mode to determine the tLD parameter. (**A**) Blue-sideband transition after sideband cooling, with the dark slit of the HG_01_ mode steered onto the ion. The fit yields a Rabi frequency of Ω_bsb_(*z* = 0) = 2π × 2.106(4) kHz. (**B**) Carrier transition, with the axial center of the HG_01_ mode shifted by $w_{\text{HG}}/\sqrt{2}\approx 0.72\;\mu m$ from the ion. The fit yields a Rabi frequency of $\Omega_{\text{car}}\left(z=w_{\text{HG}}/\sqrt{2}\right)=2\pi \times 119.46\left(24\right)$ kHz. For both measurements, around 6 μW of the 435-nm laser is incident on the ion.

As shown in Fig. 8, the experimental measurements yield a tLD parameter of $\eta_{⊥}^{\left(\text{exp}\right)}=0.02139\left(6\right)$, in good agreement with the value predicted from the beam size. Moreover, we can compare the tLD parameter with the conventional longitudinal Lamb-Dicke (lLD) parameter, defined as

$$\eta_{∥}=\frac{2\pi }{\lambda }\Delta x$$ (10)

where Δ*x* is the spatial size of the wave packet along the radial direction. For a radial trap frequency of around 2π × 3 MHz, the lLD parameter is around 0.064, about three times larger than the tLD parameter.

### Estimation of average phonon number

In trapped-ion systems, we can extract the phonon distribution of a given motional mode from the time evolution of the qubit state under sideband interactions. Specifically, we assume that the ion is prepared in the state

$$\rho =|0\rangle \langle 0|⊗\rho^{\left(m\right)}$$ (11)

where ρ^(m)^ is the density matrix of a single motional mode, and is then driven on the blue-sideband transition by

$$H_{\text{bsb}}=\Omega_{\text{bsb}}\left(\sigma_{+}\;a^{†}+\sigma_{-}a\right)$$ (12)

where *a* (*a*^†^) is the anihilation (creation) operator of the motional mode. Under this situation, the time evolution of the probability of finding the qubit in state ∣1⟩, denoted as *P*_1_, can be analytically solved as (*65*)

$$P_{1}\left(t\right)=\sum_{n}\rho_{n,n}^{\left(m\right)}\frac{1-\text{cos}\left(2\Omega_{n,n+1}t\right)}{2}$$ (13)

where $\rho_{n,n}^{\left(m\right)}={\left\langle {n|}_{m}\rho^{\left(m\right)}|n\right\rangle }_{m}$ are the diagonal elements of the motional-state density matrix in the Fock-state basis $\left\{|n\rangle {}_{m},n=0,1,2\ldots \right\}$. In our case, the phonon-number–dependent Rabi frequency $\Omega_{n,n+1}$ can be approximated as $\Omega_{n,n+1}\approx \sqrt{n+1}\Omega_{\text{bsb}}$.

If the motional mode is prepared in a thermal state, the diagonal elements $\rho_{n,n}^{\left(m\right)}$ follow the standard thermal distribution

$$\rho_{n,n}^{\left(m\right)}=\frac{{\bar{n}}^{n}}{{\left(1+\bar{n}\right)}^{n+1}}$$ (14)

where $\bar{n}$ is the average phonon number characterizing the thermal excitation of the motional mode.

In the experiment, the average phonon number of the axial motional mode after sideband cooling is extracted by maximum-likelihood fitting of the blue-sideband time-evolution data using

$$P_{1}^{\left(\text{fit}\right)}\left(t\right)=\sum_{n}\frac{{\bar{n}}^{n}}{{\left(1+\bar{n}\right)}^{n+1}}\frac{A-B\text{cos}\left(2\sqrt{n+1}\Omega_{\text{bsb}}t\right)}{2}$$ (15)

with *A*, *B*, Ω_bsb_, and $\bar{n}$ as fitting parameters. Hereby we can estimate the average phonon number after the CSC.
