# Supplementary material for: Scalable entangling gates on ion qubits via structured light addressing
Source: Sci Adv. 2026 Apr 1;12(14):eaec0392. doi: 10.1126/sciadv.aec0392 (PMC13041766; doi:10.1126/sciadv.aec0392)
Supplement: Supplementary file 1 — Supplementary Text Figs. S1 and S2 Table S1 [file sciadv.aec0392_sm.pdf]

Science Advances
AAAS

Supplementary Materials for

Scalable entangling gates on ion qubits via structured light addressing

Xueying Mai *et al.*

Corresponding author: Junhua Zhang, zhangjunhua@iqasz.cn; Yao Lu, luyao.phy@gmail.com

Sci. Adv. **12**, eaec0392 (2026)
DOI: 10.1126/sciadv.aec0392

This PDF file includes:

Supplementary Text
Figs. S1 and S2
Table S1

Supplementary Text

Entanglment in three-ion chain

In the maintext we presents the detailed data of Bell-state fidelity for the (1,3) ion pair, Fig. S1(A-B) shows supplementary data for nearest-neighbor (1,2) and (2,3) pairs. The measured fidelities for these adjacent pairs are systematically lower than those of the outermost (1,3) pair. As mentioned in the maintext, we attribute the fidelity difference to the long-tail induced field amplitude crosstalk for structured beams generated by $0-\pi$ phase plate. This amplitude crosstalk induces the off-resonant carrier coupling that degrade gate performance, which is also present in the two-ion chain case.

In Fig. S1C, we benchmark the $XX_{1,3}(\pi/4)$ gate mediated by the breathing mode, implemented with a detuning of $2\pi\times 1/120$ kHz from the breathing mode frequency due to limited 435 nm laser power. Thus the gate time increases to 140 μs (including 20 μs $\sin^2$-ramping up/down). The breathing mode's inhomogeneous motional pattern suppresses heating effects and naturally decouples the center ion, reducing gate errors from both motional heating and gradient crosstalk compared to the COM-mode-mediated case. A complete error budget is provided in Table S1.

For the error budget of the COM-mode-mediated gate shown in Table 1, the spectator-mode error mainly arises from unclosed phase-space trajectories of the non-COM modes. Taking into account the thermal occupation of these modes after only Doppler cooling, the resulting spectator-mode error is still below 10^{-6}.

In contrast, for the breathing-mode-mediated gate, the spectator-mode error is dominated by heating of the COM mode, even though the COM mode is only weakly excited during the gate. Numerical simulations show that, given our measured heating rate and gate duration in the three-ion case, this contribution can reach 4.3×10^{-6}, which is much larger than the error arising from unclosed phase-space trajectories. Nevertheless, it remains sufficiently small that it does not make a substantial contribution to the total gate error. The empirical formula given in Reference (*43*),

$$\epsilon_{\rm h} \approx \dot{\bar{n}}\langle|\alpha_{\rm COM}|^2\rangle\tau_{\rm g}, \tag{S1}$$

provides a consistent estimate of the heating-induced error, $\epsilon_{\rm h} \approx 4.1\times10^{-6}$. Here $\dot{\bar{n}}$ is the heating rate of the COM mode, $\tau_{\rm g}$ is the gate time, $\langle|\alpha_{\rm COM}|^2\rangle\tau_{\rm g} = \int_0^{\tau_{\rm g}} |\alpha_{\rm COM}(t)|^2 {\rm d}t$ is the average displacement of the COM mode in the phase space.

In our error budget decomposition, we employ the Lindblad master equation shown in Equation (S2) to numerically analyze the contributions from all relevant error sources. The effective qubit decoherence arising from laser phase noise (T_z), the finite lifetime of the $\mathrm{D}_{3/2}$ manifold ($T_{\mathrm{D}_{3/2}}$), and heating of the COM motional mode ($\dot{\bar{n}}$) are included. The strength of each dissipative channel is independently characterized. The evolution Hamiltonian $H(t)$ includes the state-dependent force applied to the targeted ions, as well as coherent error sources such as crosstalk and coupling to spectator motional modes.

$$
\begin{aligned}
\dot{\rho} = &- \mathrm{i}\,[H(t), \rho] \\
&+ \frac{1}{2T_\mathrm{z}} \sum_j \left[\sigma_\mathrm{z}^{(j)} \rho \sigma_\mathrm{z}^{(j)} - \frac{1}{2} \left(\sigma_\mathrm{z}^{(j)} \sigma_\mathrm{z}^{(j)} \rho + \rho \sigma_\mathrm{z}^{(j)} \sigma_\mathrm{z}^{(j)} \right) \right] \\
&+ \frac{1}{T_{\mathrm{D}_{3/2}}} \sum_j \left[\sigma_-^{(j)} \rho \sigma_+^{(j)} - \frac{1}{2} \left(\sigma_+^{(j)} \sigma_-^{(j)} \rho + \rho \sigma_+^{(j)} \sigma_-^{(j)} \right) \right] \\
&+ \dot{\bar{n}} \left[a_1{}^\dagger \rho a_1 - \frac{1}{2} \left(a_1 a_1{}^\dagger \rho + \rho a_1 a_1{}^\dagger \right) \right] \\
&+ \dot{\bar{n}} \left[a_1 \rho a_1{}^\dagger - \frac{1}{2} \left(a_1{}^\dagger a_1 \rho + \rho a_1{}^\dagger a_1 \right) \right] .
\end{aligned}
\tag{S2}
$$

Pulse optimization for entangling gates in a 100-ion chain

Here, we consider an ion chain consisting of N ions. The frequencies of the axial collective motional modes are denoted as $\{\nu_m, m = 1, 2, 3, ..., N\}$, and the corresponding creation and annihilation operators for the m-th motional mode are a_m and $a_m^\dagger$, respectively. By addressing the j-th and j'-th ion qubits and applying state-dependent forces, the interaction Hamiltonian can be written as below,

$$
H(t) = \sum_{k=j,j'} \sum_m \eta_\perp b_{k,m} \Omega_k^{(\mathrm{G})}(t) (a_m e^{i\delta_m t} + a_m^\dagger e^{-i\delta_m t}) \sigma_\mathrm{x}^{(k)} . \tag{S3}
$$

Here $\Omega_k^{(\mathrm{G})}$ denotes the effective carrier Rabi frequency on the k-th ion driven by the fundamental Gaussian mode. Without loss of generality, we set $\Omega_j^{(\mathrm{G})}(t) = \Omega_{j'}^{(\mathrm{G})}(t) = \Omega(t)$, where $\Omega(t)$ can be varied in time to implement optimized control (amplitude modulation (*13*)). The coefficients $\{b_{k,m}\}$ describe the participation of the k-th ion in the m-th motional modes, and $\delta_m = \mu - \nu_m$ is the detuning between the beatnote of the bichromatic fields and the frequency of the m-th motional modes.

Under the Hamiltonian above, the unitary evolution of the j-th and j'-th qubits is given by,

$$U(\tau) = \exp\left[\sum_{k=j,j'}\sum_{m}(\alpha_{k,m}(\tau)a_m^{\dagger} - \alpha_{k,m}^{*}(\tau)a_m)\sigma_{\mathrm{x}}^{(k)} - i\phi_{j,j'}(\tau)\sigma_{\mathrm{x}}^{(j)}\sigma_{\mathrm{x}}^{(j')}\right]. \tag{S4}$$

Here, $\alpha_{k,m}(\tau)$ is the phase-space-trajectory (PST) of the m-th motional mode contributed by the k-th ion, and $\phi_{j,j'}(\tau)$ is the accumulated geometric phase contributed by ions j and j'. These two factors can be expressed as,

$$\alpha_{k,m}(\tau) = -i\eta_{\perp}b_{k,m}\int_0^{\tau}\Omega(t)e^{-i\delta_m t}dt, \tag{S5}$$

$$\phi_{j,j'}(\tau) = -2\eta_{\perp}^2\sum_m b_{j,m}b_{j',m}\int_0^{\tau}dt_2\int_0^{t_2}\Omega(t_2)\Omega(t_1)\sin\left[\delta_m(t_2-t_1)\right]dt_1. \tag{S6}$$

In general, to realize a high-fidelity entangling gate $\mathrm{XX}_{j,j'}(\pi/4)$ between two qubits at a gate time τ_{g}, the pulse must be optimized such that the following constraints are satisfied,

$$\sum_{k=j,j'}\sum_{m}\left|\alpha_{k,m}(\tau_{\mathrm{g}})\right|^2 = 0, \tag{S7}$$

$$\phi_{j,j'}(\tau_{\mathrm{g}}) = \pi/4. \tag{S8}$$

For entangling gates mediated by the radial motion, nearly all collective modes must be included due to the dense radial spectrum, leading to complex pulse control.

In contrast, for entangling gates mediated by the axial motion, only a few motional modes need to be considered in pulse optimization due to the sparse axial spectrum. Therefore, the optimization constraint of Equation (S7) can be modified to be

$$\sum_{k=j,j'}\sum_{m\in\text{chosen set}}\left|\alpha_{k,m}(\tau_{\mathrm{g}})\right|^2 = 0, \tag{S9}$$

where the size of the chosen set is believe to be much smaller than the length of ion chain. The cumulative gate error from all motional modes can be expressed as (*20, 43*),

$$\epsilon_{j,j'} = \sum_{k=j,j'}\sum_{m}\left|\alpha_{k,m}(\tau_{\mathrm{g}})\right|^2 \tag{S10}$$

To evaluate the number of motional modes required for optimized control using axial motion, we consider a chain of 100 ions. The axial trap frequency is set to $2\pi \times 0.051$ MHz, corresponding to a minimal ion spacing of 3 μm. The numerically simulated motional spectrum is illustrated in

Fig. S2A. In our pulse design, the gate time is set to 220 μs, with 20-μs $\sin^2$-ramping periods at both ends. The 180-μs intermediate window is divided into 40 segments, whose amplitudes serve as degrees of freedom in the optimization to satisfy the constraint in Equation (S9).

To achieve arbitrary connectivity, we each select the non-COM modes ν_3, ν_4 and ν_5 as the primary entanglement mediators. The beatnote of the bichromatic fields is set $2\pi\times5.6$ kHz below the frequency of the primary mediator, held for all the pulse cases below. During pulse optimization, the two collective motional modes closest to the primary mediator are included in the set. Additionally, the COM mode (ν_1) is always included in the optimization to minimize errors arising from its thermal excitation.

In Fig. S2B, we show the normalized optimized pulse when the third motional mode ν_3 is utilized as the primary entanglement mediator, with the set of $\{\nu_1, \nu_2, \nu_3, \nu_4\}$ included in the optimization. Using the same optimized pulse shape, $\mathrm{XX}_{j,j'}(\pi/4)$ gates can be implemented on different ion pairs (j, j') simply by adjusting the overall coupling strength. Based on the corresponding coupling strength, the gate infidelity for each pair is then evaluated using Equation (S10), taking into account contributions from all motional modes. As shown in Fig. S2C, the gate errors from all motional modes can be suppressed below 10^{-3} for approximately 97% of ion pairs. The remaining pairs exhibit higher cumulative errors because the ions involved are weakly coupled to the selected third motional mode.

We further design pulses using modes $m = 4$ and 5 as the primary mediators. The resulting optimized pulse shapes and the corresponding gate errors for all ion pairs are shown in Fig. S2(D-G), By combining the three pulse shapes associated with different primary mediators, arbitrary ion-pair entanglement can be realized in a 100-ion chain, with gate errors induced by residual qubit-motion couplings below 10^{-3}. Further suppression of this error can be achieved by extending the gate duration or including additional modes into the optimization.

In the 100-ion example, we use non-COM modes ν_3 to ν_5 as the primary entangling mediators to achieve arbitrary long-range entangling capability. This keeps the COM mode nearly decoupled. Using non-COM modes that are farther away would further suppress infidelity arising from COM-mode heating. In this case, more non-COM modes must be included as primary entangling mediators to cover different parts of the connectivity. Meanwhile, due to the nearly linear dispersion of the axial spectrum, the optimization complexity does not increase when choosing non-COM

modes farther away from the COM mode as mediators.

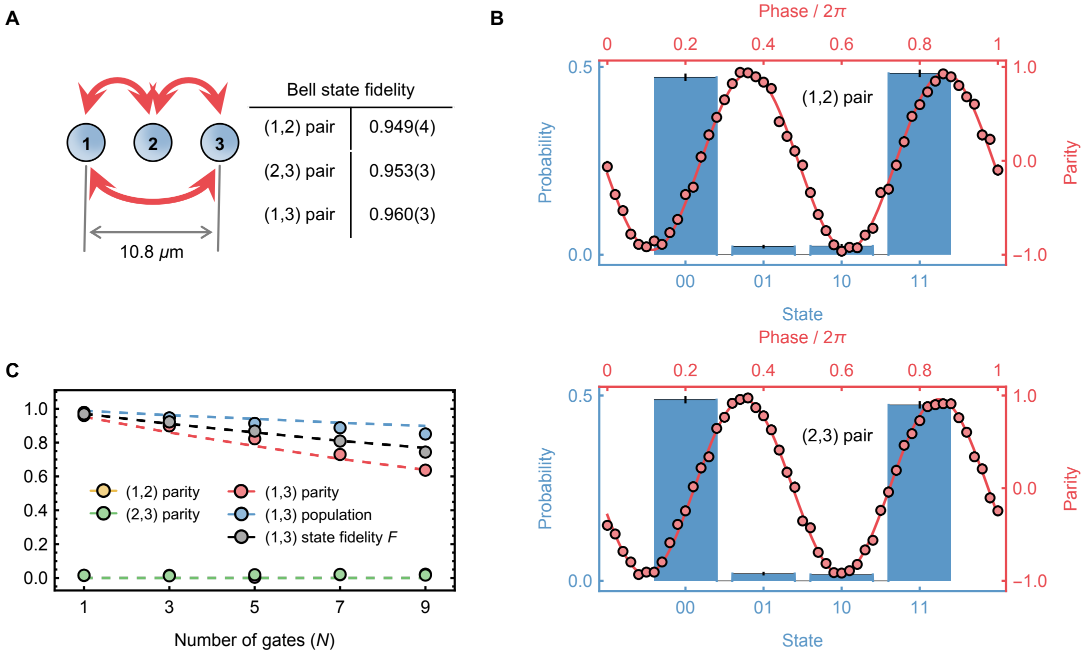

Fig. S1: **Entanglement generation in the three-ion chain.** (**A**) Measured Bell-state fidelities for all ion pairs. (**B**) Bell-state characterization for (1,2) (top) and (2,3) (bottom) pairs. The blue histogram displays the state populations, while the parity oscillation contrast quantifies off-diagonal coherence. (**C**) Breathing-mode-mediated Bell-state fidelity versus gate repetitions for the (1,3) pair. Colored markers indicate experimental results, and dotted lines show numerical simulations accounting for experimental error sources.

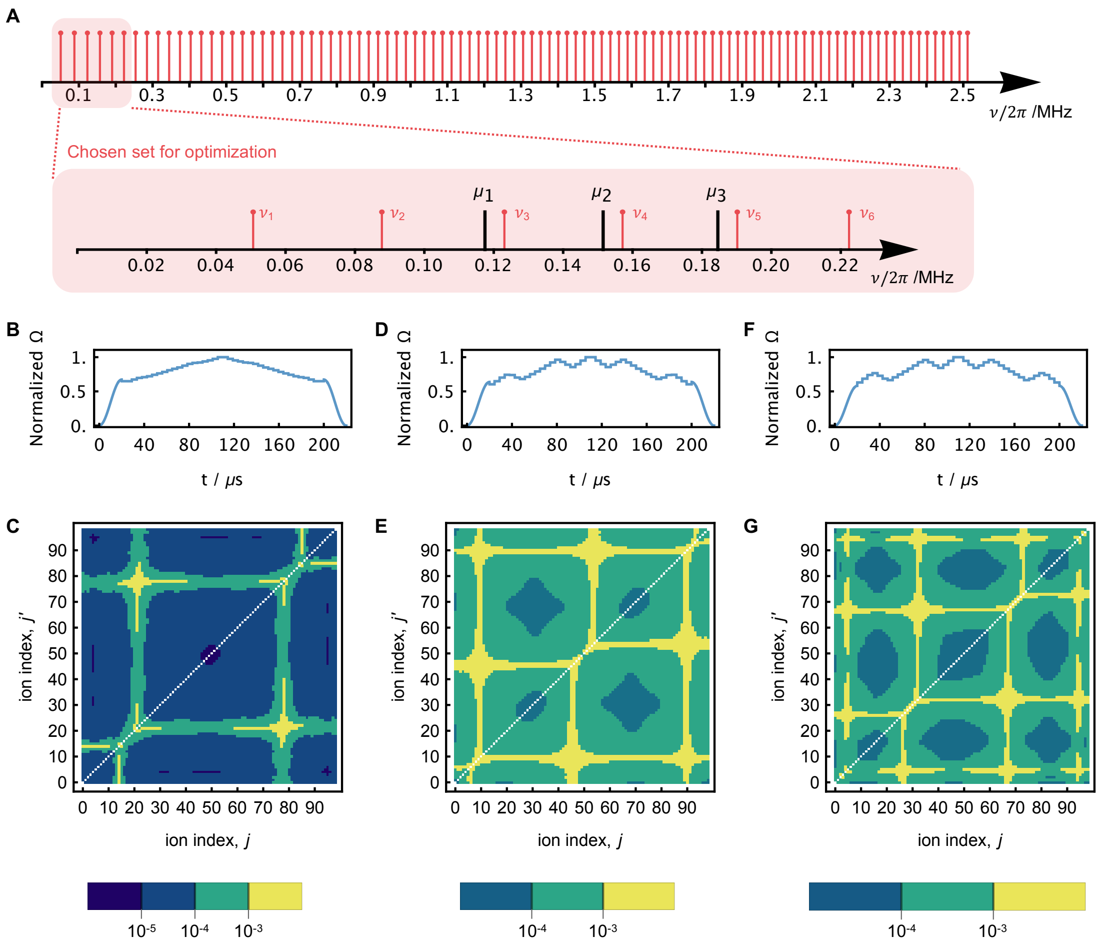

Fig. S2: **Optimized control for entangling gates in a 100-ion chain.** (**A**) Axial motional spectrum for a 100-ion chain, with an axial trap frequency of $2\pi \times 0.051$ MHz. The non-COM modes ν_3, ν_4 and ν_5 are chosen as the primary entanglement mediators to enable connectivity across all qubit pairs. During pulse optimization, a subset of motional modes from ν_1 to ν_6 is included to ensure that their trajectories in phase space are properly closed. (**B - C**) Optimized pulse using ν_3 as the entanglement mediator, and the gate errors raised by the residual qubit-motion couplings across all qubit pairs. The set of $\{\nu_1, \nu_2, \nu_3, \nu_4\}$ is included in the optimization (**D - E**) Optimized pulse using ν_4 as the entanglement mediator and corresponding gate errors. The set of $\{\nu_1, \nu_3, \nu_4, \nu_5\}$ is included in the optimization (**F - G**) Optimized pulse using ν_5 as the entanglement mediator and corresponding gate errors. The set of $\{\nu_1, \nu_4, \nu_5, \nu_6\}$ is included in the optimization

Table S1: **Error budget for the entangling gate $\mathrm{XX}_{1,3}(\pi/4)$ mediated by breathing mode.** The left column summarizes the error sources considered to contribute to the overall gate error. The right column presents numerically estimated contributions, simulated using independently characterized strengths for each error channel. The experimental Bell-state infidelity and the per-gate error are obtained from Fig. S1C.

Error source	Simulated error
Laser dephasing	2.46×10^{-2}
$^2D_{3/2}$ lifetime	0.24×10^{-2}
Pointing fluctuation	$< 1 \times 10^{-3}$
Spectator modes	4.3×10^{-6}
Simulation sum	2.7×10^{-2}
Experimental Bell-state infidelity	$2.9(3) \times 10^{-2}$
Experimental per-gate error	$2.8(1) \times 10^{-2}$
